# Identification of a novel cellular senescence-related signature for the prediction of prognosis and immunotherapy response in colon cancer

**DOI:** 10.3389/fgene.2022.961554

**Published:** 2022-08-04

**Authors:** Longfei Dai, Xu Wang, Tao Bai, Jianjun Liu, Bo Chen, Ting Li, Wenqi Yang

**Affiliations:** Department of General Surgery, The First Affiliated Hospital of Anhui Medical University, Hefei, China

**Keywords:** colon cancer, cellular senescence, prognosis, immunotherapy, signature

## Abstract

The study was conducted to construct a cellular senescence-related risk score signature to predict prognosis and immunotherapy response in colon cancer. Colon cancer data were acquired from the Gene Expression Omnibus and The Cancer Genome Atlas databases. And cellular senescence-related genes were obtained from the CellAge database. The colon cancer data were classified into different clusters based on cellular senescence-related gene expression. Next, prognostic differential genes among clusters were identified with survival analysis. A cellular senescence-related risk score signature was developed by performing the LASSO regression analysis. Finally, PCA analysis, t-SNE analysis, Kaplan-Meier survival analysis, ROC analysis, univariate Cox regression analysis, multivariate Cox regression analysis, C-index analysis, meta-analysis, immune infiltration analysis, and IPS score analysis were used to evaluate the significance of the risk signature for predicting prognosis and immunotherapy response in colon cancer. The colon cancer data were classified into three clusters. The patients in cluster A and cluster B had longer survival. A cellular senescence-related risk score signature was developed. Patients in the low-risk score group showed a better prognosis. The risk score signature could predict colon cancer patients’ prognosis independently of other clinical characteristics. The risk score signature predicted the prognosis of colon cancer patients more accurately than other signatures. Patients in the low-risk score group showed a better response to immunotherapy. The opposite was true for the high-risk score group. In conclusion, the cellular senescence-related risk score signature could be used for the prediction of prognosis and immunotherapy response in colon cancer.

## Introduction

Nowadays, colon cancer has high morbidity and mortality all over the world, which seriously threatens human life (Sung et al., 2021). Surgical resection is preferred for early-stage colon cancer, while systemic chemotherapy is the main treatment for advanced colon cancer (Chakrabarti et al., 2020; Body et al., 2021). However, the effectiveness of chemotherapy for partial colon cancer patients is often unsatisfactory due to the emergence of drug resistance (Azwar et al., 2021). In recent years, immunotherapy has brought new hope for the treatment of cancer (Lichtenstern et al., 2020). Immunotherapy drugs (PD1/PD-L1 blocker and CTLA-4 blocker) have been shown to improve the prognosis of colon cancer patients (Yaghoubi et al., 2019; Ben et al., 2021). Unfortunately, the prognosis and immunotherapy responses of different colon cancer patients are significantly differentiated due to the existence of tumor heterogeneity (Marisa et al., 2021; Guo et al., 2022). Therefore, it is particularly important to distinguish between patients with colon cancer who show a better prognosis and immunotherapy response.

Cellular senescence, the permanent cessation of cell proliferation, is thought to be able to prevent the development and metastasis of tumor cells (Calcinotto et al., 2019; Di Micco et al., 2021). However, recent studies have shown that senescent cancer cells promote tumorigenesis in neighboring cells through the release of SASP (Prieto and Baker, 2019). Demirci et al. revealed that it was the Jekyll and Hyde nature of cancer cell senescence (Demirci et al., 2021). Furthermore, cellular senescence had been demonstrated to be a potential target for cancer in clinical therapy (Prasanna et al., 2021; Wang et al., 2022). Lin et al. found that cellular senescence was important in the prognosis and immunotherapy of lung cancer (Lin et al., 2021). And Zhou et al. demonstrated that cellular senescence was a potential marker of prognosis and therapeutic outcome in gastric cancer (Zhou et al., 2022). However, the role of cellular senescence in the prognosis and immunotherapy of colon cancer is not well understood.

In this study, we aimed to investigate the significance of cellular senescence in the prognosis and immunotherapy of colon cancer. Meanwhile, a cellular senescence-related risk score signature was constructed to distinguish patients with a better prognosis and immunotherapy response.

## Methods

### Acquisition of colon cancer information and cellular senescence-related genes

Transcriptome information, clinical information, and mutation information were acquired from The Cancer Genome Atlas (TCGA) database (https://portal.gdc.cancer.gov/). Next, the gene ID from the transcriptome information was converted into gene names to obtain TCGA expression data. Then the FPKM value of TCGA expression data was converted into the TPM value. The survival time, survival status, age, gender, pathological TNM stage, pathological T-stage, pathological N-stage, and pathological M-stage were extracted from the clinical information. The platform file GPL570 and probe matrix GSE39582 were also downloaded from the Gene Expression Omnibus (GEO) database (https://www.ncbi.nlm.nih.gov/geo/). The probe matrix was transformed into a gene matrix by finding the correspondence between the probe matrix and gene names based on the platform file information. The copy number data of colon cancer were obtained from UCSC Xena (http://xena.ucsc.edu/). Finally, the TCGA expression data and GEO expression data were merged to obtain the expression of genes in the merged data. Cellular senescence-related genes were acquired from the CellAge database (Supplementary Table S1). The expression of cellular senescence-related genes was extracted from the merged data. The workflow chart was visualized in Supplementary Figure S1.

### Clusters based on cellular senescence-related gene expression

The “ConsensusClusterPlus” package was used to perform a Consensus Clustering analysis on the merged data. The principal component analysis (PCA) was performed to validate the accuracy of distinguishing different clusters based on cellular senescence-related gene expression. Kaplan-Meier survival analysis was performed with the “survival” and “survminer” packages. The heatmap was plotted by using the “pheatmap” package. The single-sample gene set enrichment analysis (ssGSEA) and gene set variation analysis (GSVA) were conducted based on the “GSEABase” and “GSVA” packages.

### Differential analysis of clusters

Differential genes (DEGs) among clusters were identified (adjusted *p*-value = 0.001). The Gene Ontology (GO) and Kyoto Encyclopedia of Genes and Genomes (KEGG) enrichment analyses were performed to explore enrichment pathways on DEGs.

### Gene clusters based on differential genes

Prognostic genes were identified by performing survival analysis with the “survival” package (filtering condition: *p*-value < 0.05). The “ConsensusClusterPlus” package was applied to conduct the Consensus Clustering analysis on DEGs among clusters. PCA analysis was performed to validate the accuracy of distinguishing different gene clusters based on DEGs among clusters. Kaplan-Meier survival analysis was applied with the “survival” and “survminer” packages. The heatmap was plotted with the “pheatmap” package.

### Constructing a risk score signature

The colon cancer data were classified into training and testing sets. The selection operator (LASSO) Cox regression analysis with 10-fold cross-validation was performed to construct a prognostic risk score signature in the training set. The testing set was used to validate the accuracy of the signature. Formula:

Risk score=∑i1(Coefi∗ ExpGenei)

“Coef”, regression coefficient; “ExpGene”, the expression of genes. The training and testing sets were divided into high- and low-risk groups based on the medium value of risk scores. Principal component analysis (PCA) and t-distributed stochastic neighbor embedding analysis (t-SNE) were applied to confirm the signature’s accuracy to distinguish between high- and low-risk score groups. The Kaplan-Meier survival curves and ROC curves were plotted with “survival”, “survminer”, and “timeROC” packages. The “ggplot2” and “pheatmap” packages were applied to plot risk status, survival status, risk histogram, and risk heatmap. Univariate and multivariate Cox regression analyses were conducted to validate whether the signature predicted colon cancer patients’ prognosis independently of other clinical characteristics. C-index curves were plotted based on the “survival”, “rms”, and “pec” packages. The “timeROC” and “survcomp” packages were used to plot ROC curves and C-index histograms for comparison of signatures. The meta-analysis was performed to investigate the heterogeneity of the risk score signature in predicting the prognosis of colon cancer patients between training and testing sets with the “meta” package. The forest diagram of the meta-analysis was drawn by using the fixed-effects model.

### Developing a nomogram

A nomogram was plotted with the “regplot”, “rms”, and “survivor” packages. The ROC curve of the nomogram was drawn based on the “timeROC” package.

### Validating the risk score signature in clinical subgroups

The Sankey diagram was plotted to illustrate the construction process of the risk score signature with the “ggalluvial” package. The “ggpubr” and “ggplot2” packages were used to plot box plots to show the differences in different risk scores across clusters and gene clusters. The heatmap and box plot were drawn to investigate differences in patients’ risk scores across clinical subgroups with the “ComplexHeatmap”, “ggpubr”, and “limma” packages. We also performed Kaplan-Meier survival analysis to further validate the application of the risk score signature in different clinical subgroups.

### The landscape of gene mutation in different risk score groups

The “maftools” package was applied to visualize the gene mutation landscape in high- and low-risk score groups.

### Exploring immunotherapy response in different risk score groups

Immune score files for colon cancer were downloaded from the Cancer Immunome Database (TCIA, https://tcia.at/). Immunotherapy analysis was performed to explore the therapy differences of IPS-CTLA4, PD1, PDL1, and PDL2 blockers in patients with different risk scores with the “ggpubr” package. The“pRRophetic_0.5. tar.gz” was acquired from the Genomics of Drug Sensitivity in Cancer (GDSC, https://www.cancerrxgene.org/). Finally, the “pRRophetic” package was used to analyze the differences in half-maximal inhibitory concentration (IC50) values between different risk score groups and to identify potential drugs for colon cancer patients.

### Validating the risk score signature

Differential expression of the signature between normal and tumor samples was investigated by performing differential analysis. Finally, we searched the Human Protein Atlas (HPA, https://www.proteinatlas.org/) database for immunohistochemical results of the signature genes. In addition, the gene mutation and copy number variant of the signature genes were analyzed.

### Statistical analysis

All scripts were run in Strawberry-Perl-5.32.1.1 and all codes were run in R 4.1.2. The colon cancer data were classified into different clusters by Consensus Clustering analysis. Then, DEGs among clusters were identified. The prognostic DEGs were identified by survival analysis. The colon cancer data were again divided into different gene clusters by Consensus Clustering analysis. Next, LASSO regression analysis was performed to construct a risk score signature. Patients were classified into high- and low-risk score groups. PCA analysis and t-SNE analysis were applied to confirm the accuracy of signature in distinguishing high and low-risk score groups. Kaplan-Meier survival analysis, ROC analysis, univariate analysis, multivariate analysis, C-index method, and meta-analysis were performed to explore the role of the signature in the prognosis of colon cancer. And the TICA algorithm and “pRRophetic” package were used to investigate the significance of the signature in therapy for colon cancer. Finally, all signature genes were performed for differential analysis and validated in the HPA database. *p*-values less than 0.05 were considered to be statistically significant.

## Results

### Clusters based on cellular senescence-related gene expression level

**FIGURE 1:**
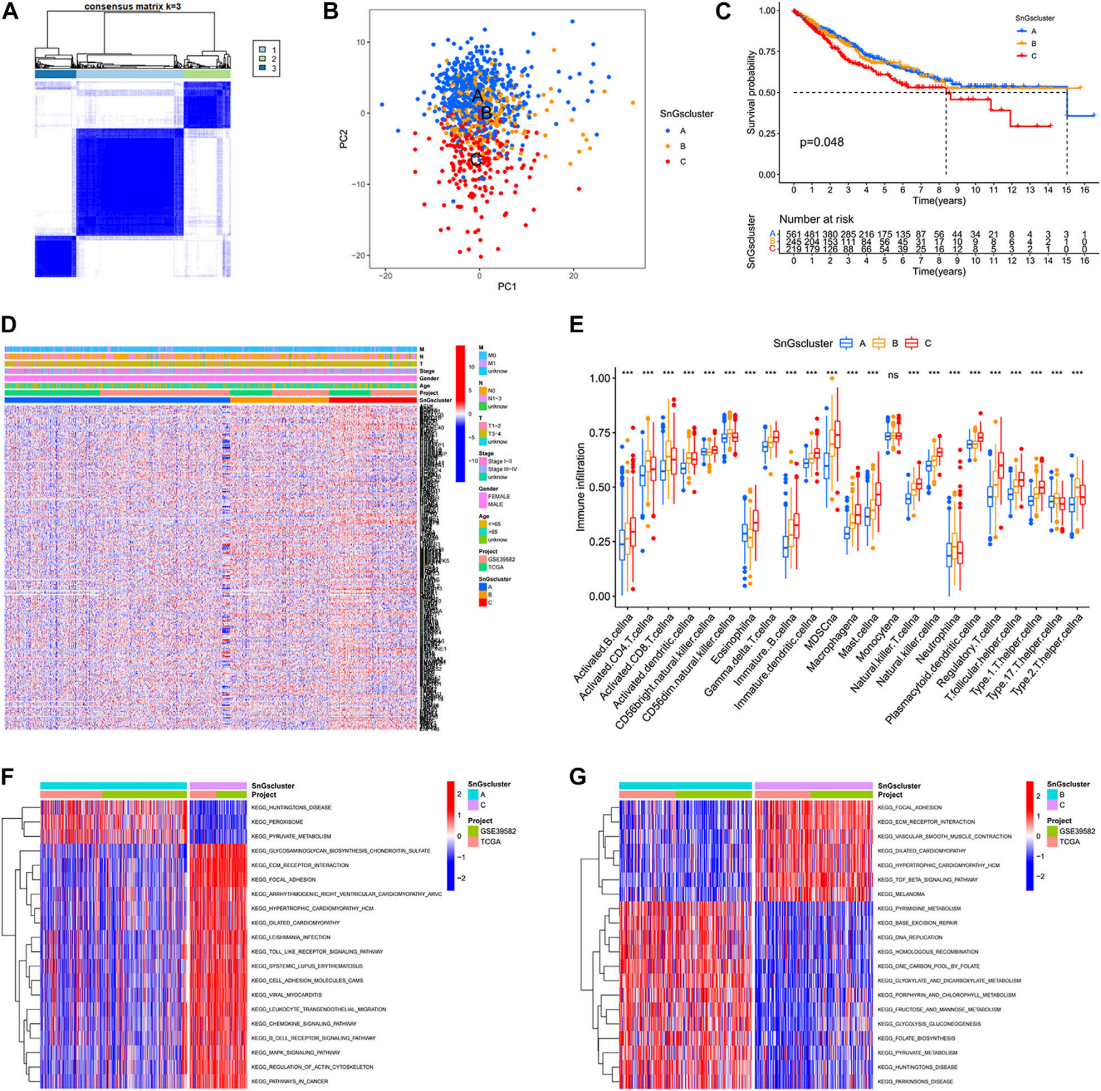
Three clusters based on cellular senescence-related gene expression levels. **(A)** Consensus Cluster Analysis. When cluster Num = 3, the relationship in the cluster was tight and the correlation between clusters was weak. **(B)** PCA plot. Blue dots represent patients in Cluster A; yellow dots represent patients in Cluster B; red dots represent patients in Cluster C. **(C)** Kaplan-Meier survival curves. The prognosis of patients was different among the three clusters, *p* = 0.048. **(D)** Heat map. Cellular senescence-related genes were upregulated in Cluster C. **(E)** Box plots. The horizontal coordinate represents immune infiltrating cells; the vertical coordinate represents immune scores; ns represents no difference in immune cell scores among the three clusters; ^∗^^∗^^∗^ *p* < 0.001. **(F)** Differential GSVA enrichment pathways between Cluster A and Cluster C. Red represents the high expression pathway and blue represents the low expression pathway. **(G)** Differential GSVA enrichment pathways between Cluster B and Cluster C. Red represents the high expression pathway and blue represents the low expression pathway.

We acquired 41 normal samples and 473 colon cancer tissue samples from the TCGA database. Another 585 colon cancer samples were obtained from the GEO database. 279 cellular senescence-related genes were acquired from the CellAge database. When cluster Num = 3, the relationship in the cluster was tight and the correlation between clusters was weak (Figure 1A). So, all samples were classified into three clusters. Other classification results were visualized in Supplementary Figure S2. In the PCA plot, red points, yellow points, and blue points were separated, which indicated that cluster A, cluster B, and cluster C could be distinguished based on the expression of cellular senescence-related genes (Figure 1B). The prognosis of patients among three clusters showed differences, with patients in cluster A and cluster B having longer survival times than cluster C (Figure 1C). We observed that cellular senescence-related genes were expressed at the lowest level in cluster A and at the highest level in cluster C (Figure 1D). And the three clusters showed no difference in the different clinical subgroups. In addition, the differences in the level of immune infiltration among the three clusters were analyzed. Interestingly, cluster C not only contained high immune cell infiltration, but also many immunosuppressive cells, such as myeloid-derived suppressor cells (MDSCs), regulatory T cells (Tregs), and macrophages (Figure 1E). It might be associated with the worse prognosis of colon cancer patients in cluster C.

We also investigated the differences in enrichment pathways among the three clusters. The significantly enriched pathways in cluster A included “PEROXISOME”, “PYRUVATE METABOLISM”, and “HUNTINGTONS DISEASE” (Figure 1F). And the predominantly enriched pathways in cluster B were “BASE EXCISION REPAIR”, “HOMOLOGOUS RECOMBINATION”, “PYRUVATE METABOLISM”, “PARKINSONS DISEASE”, and “HUNTINGTONS DISEASE” (Figure 1G). While“GLYCOSAMINOGLYCAN BIOSYNTHESIS CHONDROITIN SULFATE”, “ECM RECEPTOR INTERACTION”, “FOCAL ADHESION”, and “TGF BETA SIGNALING PATHWAY” were significantly enriched in cluster C. The KEGG pathways in cluster A and cluster B were mainly involved in tumor suppression processes, while the KEGG pathways in cluster C were associated with tumorigenesis and metastasis.

### 2334 differential genes among three clusters

To further investigate the differences among the three clusters, we identified 2334 DEGs in cluster A, cluster B, and cluster C (Supplementary Figure S3A and Supplementary Table S2). And the enrichment pathways for DEGs were visualized in Supplementary Figure S3B,C. The results of the GO enrichment analysis showed that “positive regulation of cell adhesion”, “leukocyte migration” and “leukocyte cell−cell adhesion” were significantly enriched in biological processes (BP); “collagen−containing extracellular matrix”, “cell−substrate junction”, and “focal adhesion” were significantly enriched in molecular function (CC); “actin-binding” and “extracellular matrix structural constituent” were significantly enriched in the cellular component (MF). “PI3K−Akt signaling pathway”, “Focal adhesion”, “Osteoclast differentiation”, “Rap1 signaling pathway”, and “Proteoglycans in cancer” were significantly enriched in KEGG. The significantly enriched pathways in DEGs were associated with tumor development and metastasis.

### Gene clusters based on prognostic DEGs

**FIGURE 2:**
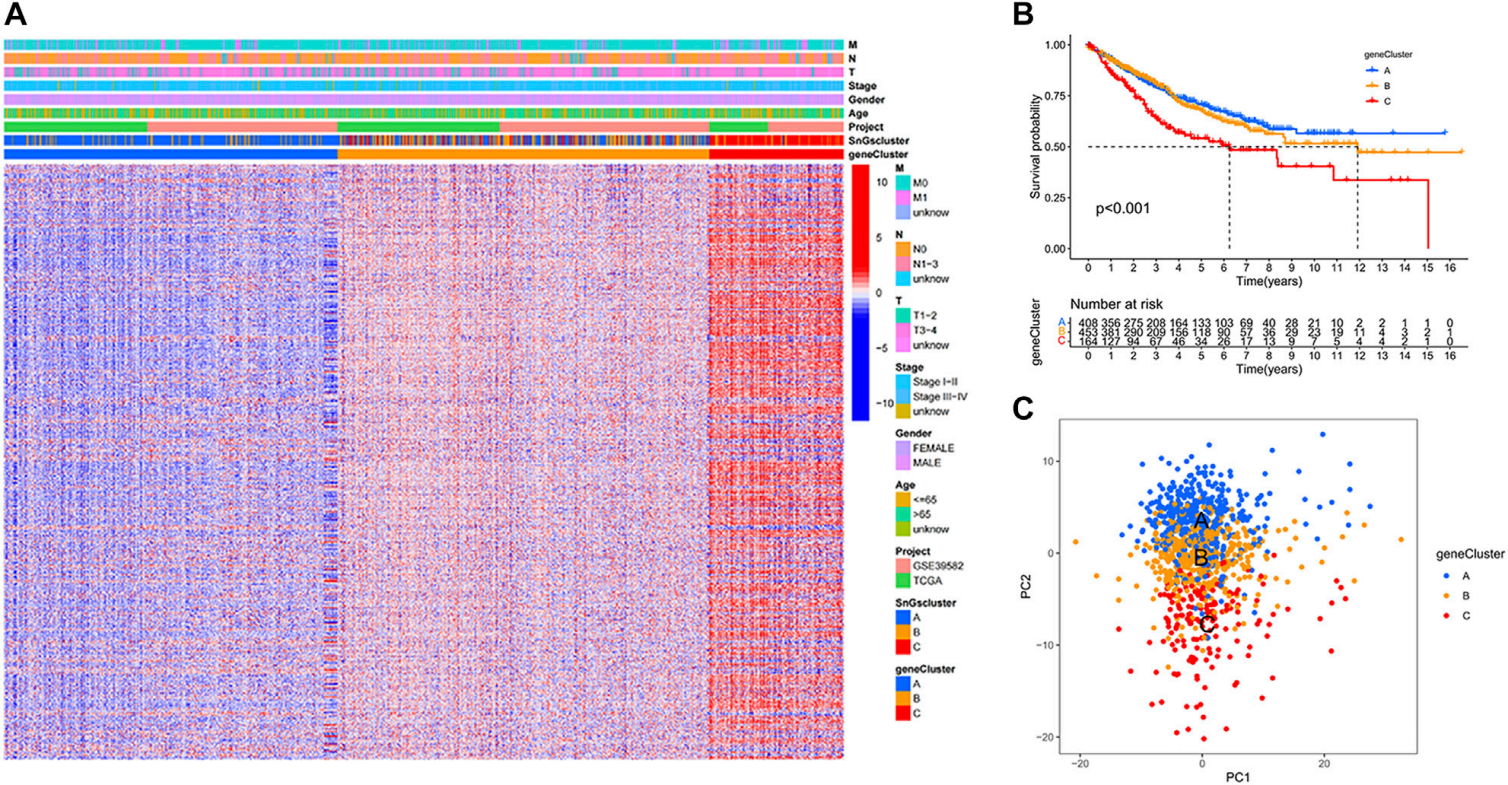
Three gene clusters based on prognostic differential gene expression levels. **(A)** Heat map. Prognostic differential genes were upregulated in gene Cluster C and down-regulated in gene Cluster A. **(B)** Kaplan-Meier survival curves. *p* < 0.001. **(C)** PCA plot. Blue dots represent patients in gene Cluster A; yellow dots represent patients in gene Cluster B; red dots represent patients in gene Cluster C.

We further studied the association of DEGs among clusters with prognosis, and 681 prognostic DEGs were identified (Supplementary Table S3). Colon cancer samples were classified into three gene clusters based on the expression of prognostic DEGs. When cluster Num = 3, the relationship in the gene cluster was tight and the correlation between gene clusters was weak (Supplementary Figure S4). The prognostic DEGs were significantly down-regulated in gene Cluster A and up-regulated in gene Cluster C (Figure 2A). Gene cluster A had the best prognosis, while gene cluster C had the worst prognosis (Figure 2B). We further validated the accuracy of classifying colon cancer samples into three gene clusters based on the expression of prognostic DEGs (Figure 2C).

### Construction and validation of a cellular senescence-related risk score signature

**FIGURE 3:**
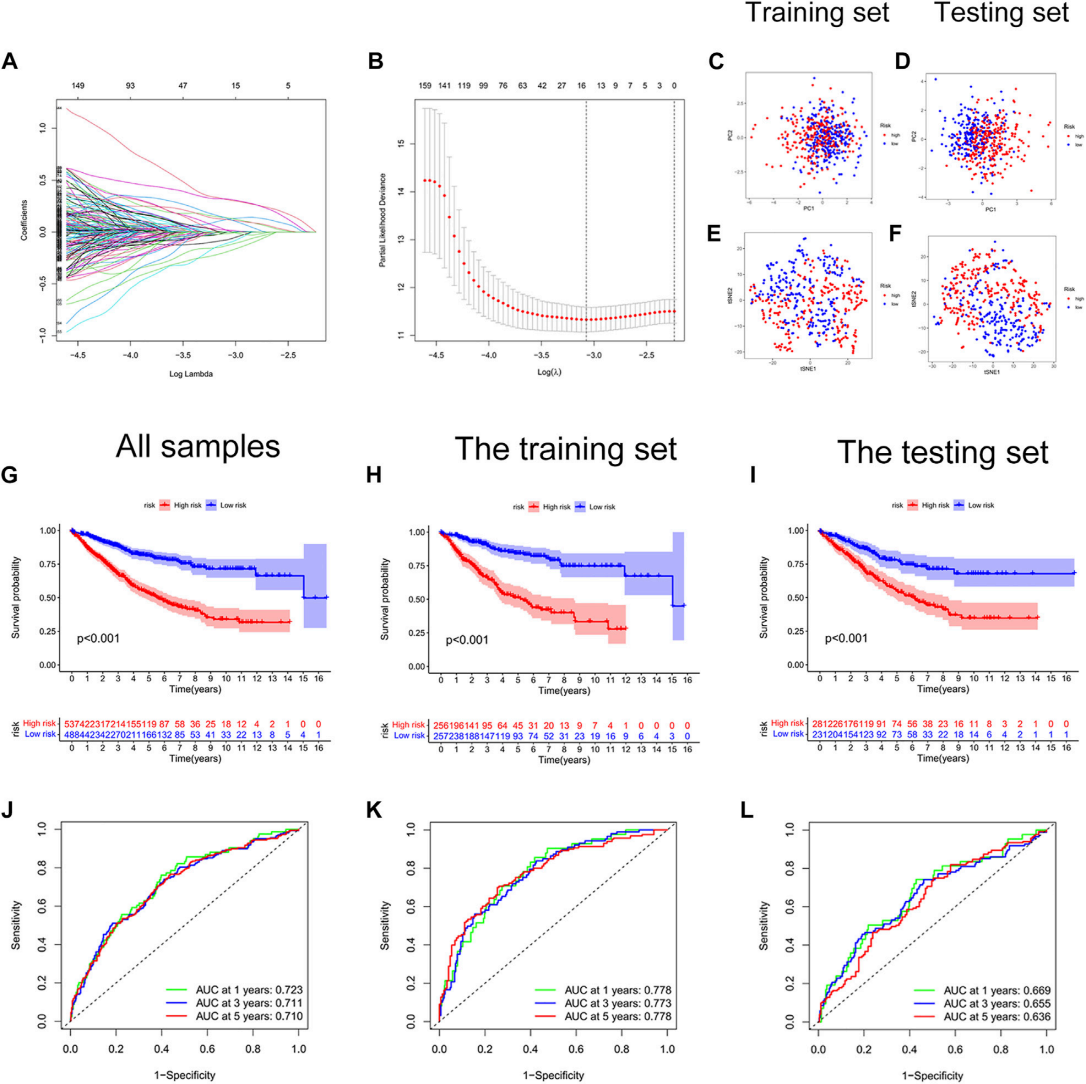
Construction of a cellular senescence-related risk score signature. **(A)** LASSO regression analysis. **(B)** Cross-validation for tuning the parameter selection. **(C)** The PCA plot of the training set. **(D)** The PCA plot of the test set. **(E)** The t-SEN plot of the training set. **(F)** The t-SEN plot of the testing set. **(G)** The K-M survival curve of all colon cancer samples, *p* < 0.001. **(H)** The K-M survival curve of the training set, *p* < 0.001. **(I)** The Kaplan-Meier survival curve of the testing set, *p* < 0.001. **(J)** The AUC values of 1-year, 3-years, and 5-years survival for all colon cancer patients were more than 0.700. **(K)** The AUC values of 1-year, 3-years, and 5-years survival for the training set were more than 0.750. **(L)** The AUC values of 1-year, 3-years, and 5-years survival for the testing set were more than 0.600.

**FIGURE 4:**
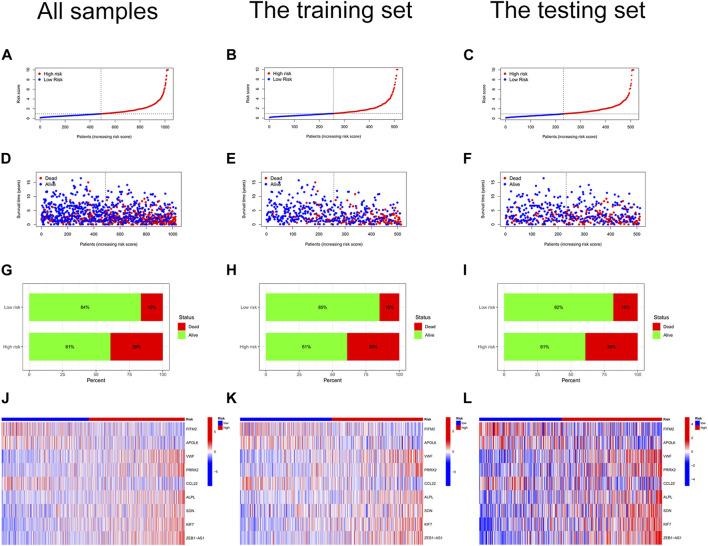
Risk curves. **(A)** Risk status plot of all colon cancer samples. The horizontal coordinate represented the ranked patients, and the risk scores of patients gradually increased from left to right; the vertical coordinate represented the risk scores. **(B)** Risk status of the training set. **(C)** Risk status of the testing set. **(D)** Survival status plot of all colon cancer samples. **(E)** Survival status plot of the training set. **(F)** Survival status plot of the testing set. **(G)** Risk histogram of all colon cancer samples. The percentage of survival patients in the low-risk score group was higher than that in the high-risk score group. **(H)** Risk histogram of the training set. **(I)** Risk histogram of the testing set. **(J)** Risk heat map of all colon cancer samples. The expression of FITM2, APOL6, and CCL22 decreased with increasing risk scores; the expression of VWF, PRRX2, ALPL, SON, KIF7, and ZEB1-AS1 increased with increasing risk scores. **(K)** Risk heat map of the training set. **(L)** Risk heat map of the testing set.

We constructed a risk score signature to predict the prognosis of colon cancer patients based on prognostic DEGs (Figures 3A,B). The training set was used to construct the risk score signature, while the testing set was applied to validate the accuracy of the signature. Formula: Risk score = FITM2 exp. * (-0.340377976707324) + APOL6 exp. * (-0.385962800820076) + VWF exp. * 0.348549751087245 + PRRX2 exp.* 0.222316119860682 + CCL22 exp. * (-0.505065627522616) + ALPL exp. * 0.427185163663466 + SON exp. * 0.403475482932549 + KIF7 exp. * 0.466822368846872 + ZEB1-AS1 exp. * 0.509278568824046. The colon cancer sample was classified into high and low-risk score groups based on the medium value of the risk score. The statistics of clinical information for the training set and the testing set were visualized in Supplementary Table S4. Red points and blue points were significantly separated in the PCA plot and the t-SNE plot, which demonstrated the accuracy of distinguishing high- and low-risk score groups based on the risk score (Figures 3C–F). We observed a better prognosis for patients in the low-risk score group (Figures 3G–I). Moreover, the prediction of patient survival at 1, 3, and 5 years was more accurate based on the signature (Figure 3J-3L). The high-risk score group had higher mortality (Figures 4A–I). The expression levels of FITM2, APOL6, and CCL22 decreased significantly with increasing risk scores, which were low-risk genes. In contrast, the expression levels of VWF, PRRX2, ALPL, SON, KIF7, and ZEB1-AS1 increased significantly with increasing risk scores, which were high-risk genes.

**FIGURE 5:**
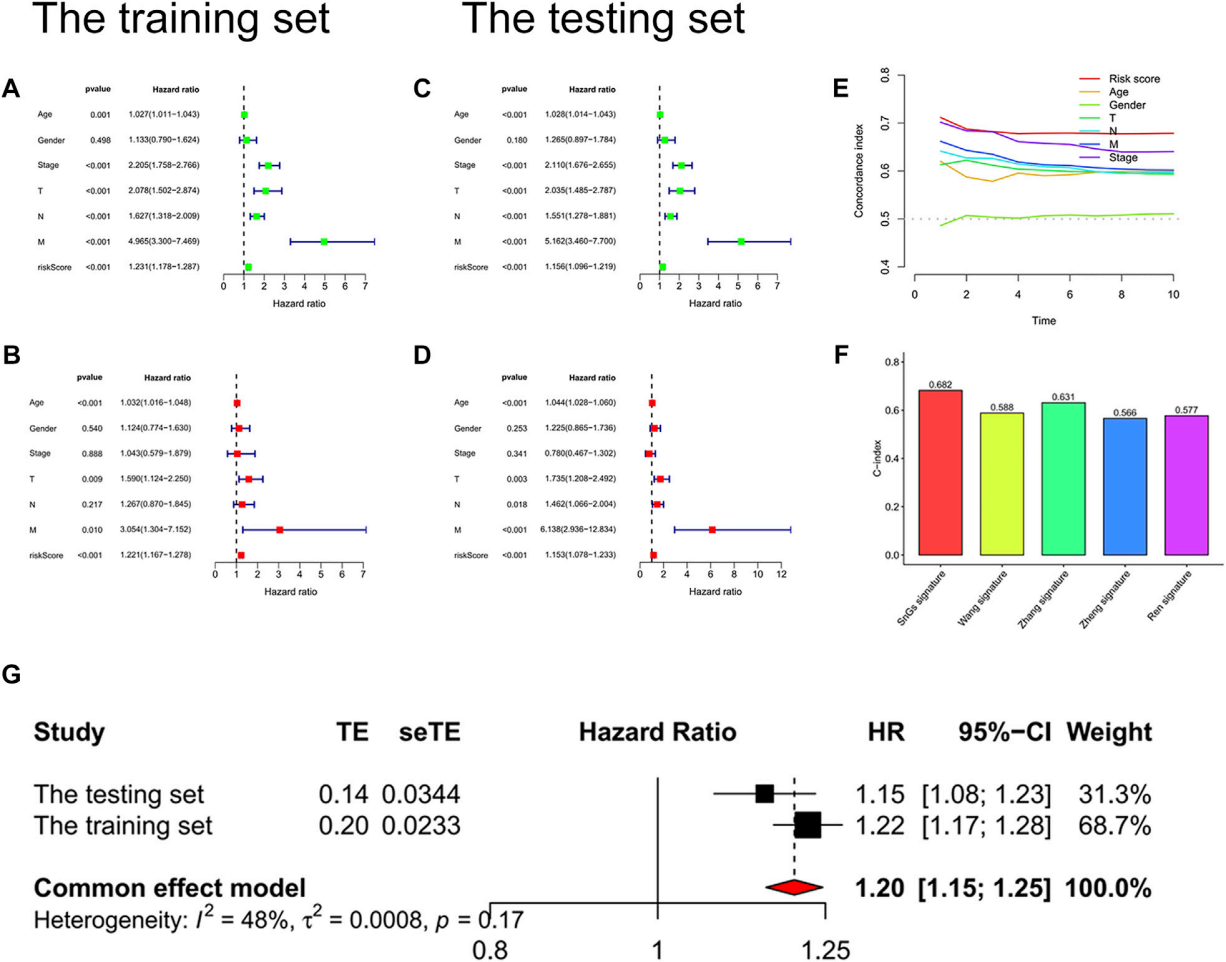
Validation of the risk score signature. **(A)** Univariate Cox regression analysis of the training set. Risk scores, *p* < 0.001. **(B)** Multivariate Cox regression analysis of the training set. Risk scores, *p* < 0.001. **(C)** Univariate Cox regression analysis of the testing set. Risk scores, *p* < 0.001. **(D)** Multivariate Cox regression analysis of the testing set. Risk scores, *p* < 0.001. **(E)** C-index curves. C-index value of risk scores was higher than other clinical characteristics (age, gender, pathological stage, T stage, N stage, and M stage). **(F)** Histogram for signature comparison with C-index method. SnGs signature had the highest C-index value compared to other signatures, AUC = 0.682. **(G)** Forest plot. The multivariate Cox regression analysis results of the training and testing sets were used to perform a meta-analysis, I2<50%.

We further confirmed the accuracy of the signature in predicting the prognosis of patients with colon cancer. *p*-values for the risk score were less than 0.001 in both univariate and multivariate Cox regression analyses, which indicated that the risk score could predict the prognosis of colon cancer patients independently of other clinical characteristics (Figures 5A–D). Moreover, the risk score predicted prognosis more accurately than other clinical characteristics, with the highest C-index value (Figure 5E). We searched online for four risk score signatures (Wang signature, Zhang signature, Zheng signature, and Ren signature) that predicted the prognosis of colon cancer (Ren et al., 2020; Zhang et al., 2020; Wang et al., 2021; Zheng et al., 2021). Surprisingly, the cellular senescence-related signature predicted the prognosis of colon cancer patients significantly better than the other four signatures, with the highest C-index value of 0.682 (Figure 5F). Supplementary Figure S5 visualized the predicted survival ROC curves and survival curves for other signatures. Furthermore, the meta-analysis showed less heterogeneity when using the signature to predict the prognosis of patients with colon cancer with I^2^ <50% (Figure 5G).

### Development of a nomogram

**FIGURE 6:**
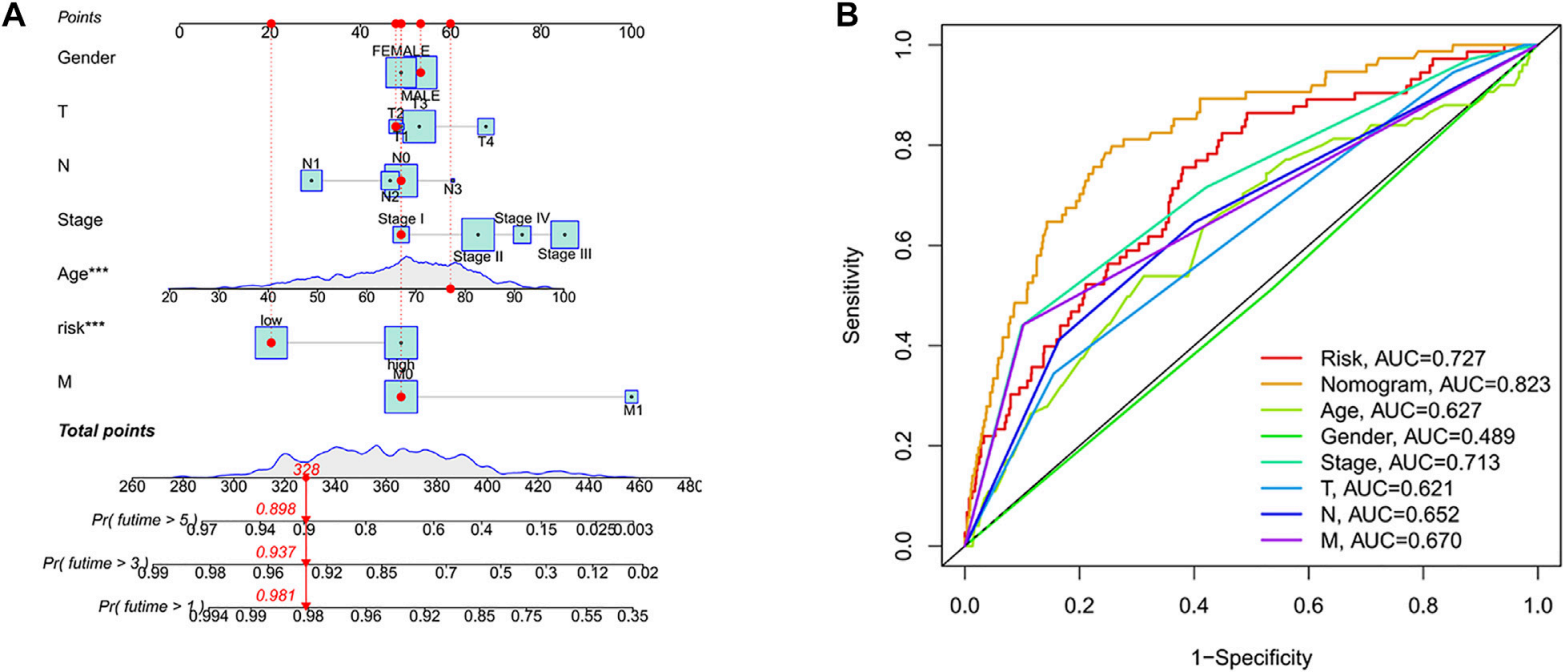
Developing a Nomogram. **(A)** Nomogram. When the total point was 328, the nomogram predicted that the 1-year survival probability of patients was more than 0.981, the 3-years survival probability was more than 0.937, and the 5-years survival probability was more than 0.898. **(B)** Nomogram ROC curve. The horizontal coordinate represented the false-positive rate expressed by 1-Specificity and the vertical coordinate represented the true-positive rate expressed by sensitivity.

A nomogram was developed to benefit clinical work in predicting 1-year, 3-years, and 5-years survival probability in patients with colon cancer. For example, when the total point was 328, the 1-year survival probability of patients was more than 0.981, the 3-years survival probability was more than 0.937, and the 5-years survival probability was more than 0.898 (Figure 6A). Moreover, we found that predicting the survival probability of colon cancer patients was significantly better than other clinical characteristics based on the nomogram, with the highest AUC value of 0.823 (Figure 6B).

### Validation of risk score signature in clinical subgroups

**FIGURE 7:**
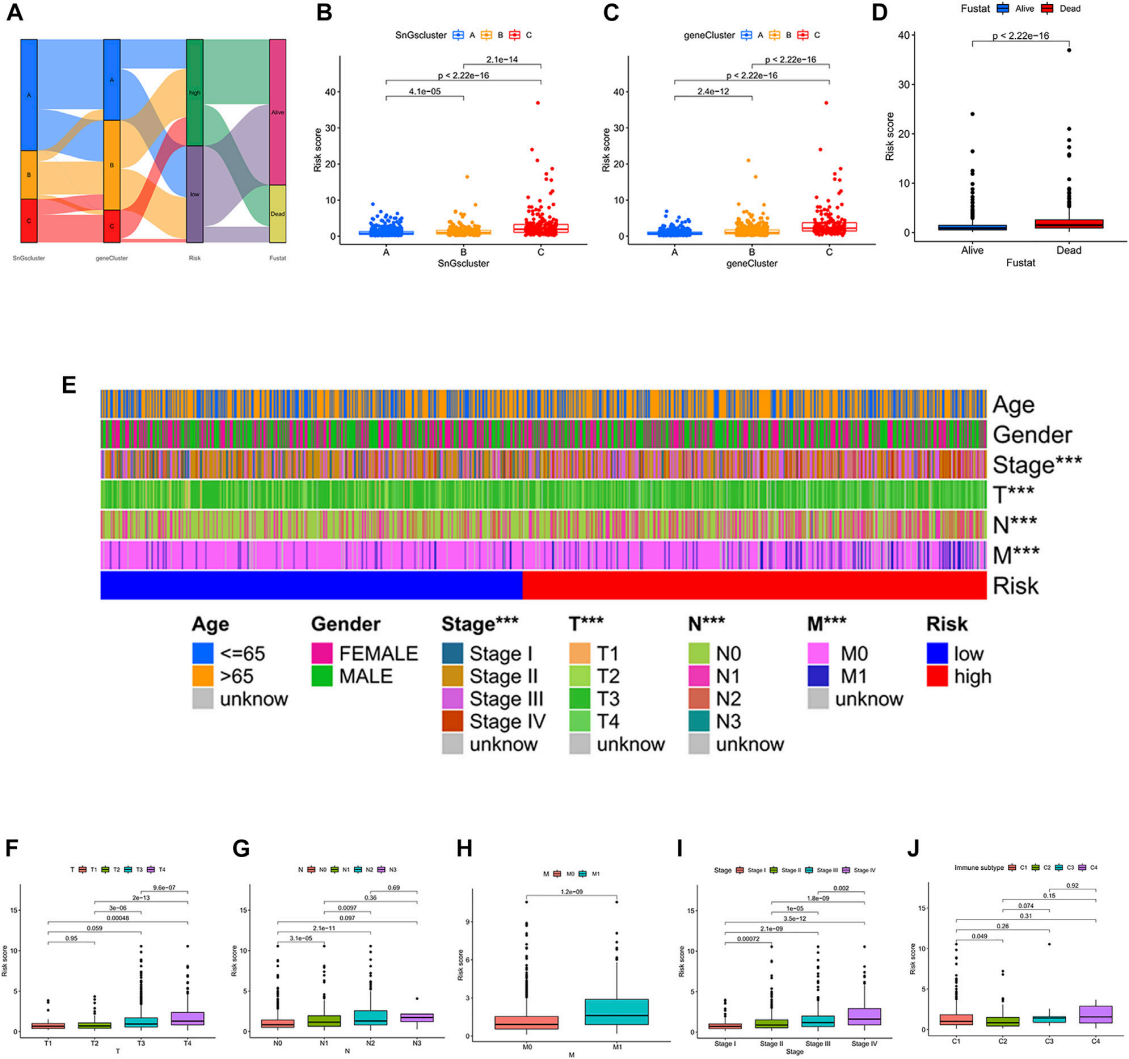
**(A)** Sankey diagram. The construction process of the risk score signature. **(B)** Box plot of risk scores for the three clusters. **(C)** Box plot of risk scores for the three gene clusters. **(D)** Box plot of risk scores for the survival status (Fustat). **(E)** Heatmap of correlation between risk and clinical characteristics. ^∗^^∗^^∗^ *p* < 0.001. **(F)** Box plot of the risk score for the T stage. **(G)** Box plot of risk scores for the N stage. **(H)** Box plot of risk scores for M stage. **(I)** Box plot of risk scores for pathological TNM stage. **(J)** A box plot of risk scores across the four immune subtypes. C1, Wound Healing; C2, IFN-gamma Dominant; C3, Inflammatory; C4, Lymphocyte Depleted.

The construction process of the signature was illustrated in Figure 7A. We further analyzed the differences in risk scores among the different clusters. Cluster A and gene Cluster A had the lowest risk scores, while cluster C and gene Cluster C had the highest risk scores (Figures 7B,C). All colon cancer patients were also classified into survival and death groups based on survival outcomes. Interestingly, patients in the survival group showed lower risk scores (Figure 7D). It further confirmed the above findings that patients in the low-risk score group had a better prognosis.

Next, we also explored whether there were differences in risk scores across clinical characteristics (Figure 7E). Unexpectedly, the risk scores showed no differences between men and women, nor between different age groups (≤65 and >65). In contrast, there were differences in risk scores among T stage (T1, T2, T3, T4), N stage (N0, N1, N2, N3), M stage (M0, M1), and pathological TNM stage (Stage I, Stage II, Stage III, Stage IV). The risk score increased gradually after T2 (Figure 7F). The risk score increased gradually after N0, except for N3 (Figure 7G). The risk score of M1 was significantly higher than that of M0 (Figure 7H). The risk score increased gradually after Stage I (Figure 7I). The immunotyping of colon cancer is classified into four subtypes, C1 (Wound Healing), C2 (IFN-gamma Dominant), C3 (Inflammatory), and C4 (Lymphocyte Depleted). Unexpectedly, there was no difference in risk scores among subtypes, except for the difference in risk scores between C1 and C2 (Figure 7J). And C2 had a lower risk score than C1.

**FIGURE 8:**
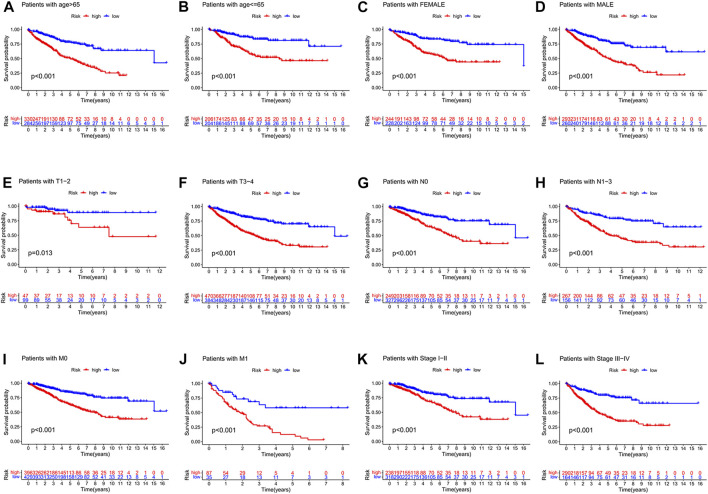
**(A-L)** visualized the risk score signature being applied to patients with different clinical subgroups, including different ages, different genders, different T-stages, different N-stages, different M-stages, and different pathological stages. **(A)** Patients with age > 65. **(B)** Patients with age ≦ 65. **(C)** Female patients. **(D)** Male patients. **(E)** Patients with stages T1-2. **(F)** Patients with stage T3-4. **(G)** Patients with stage N0. **(H)** Patients with stages N1-3. **(I)** Patients with stage M0. **(J)** Patients with stage M1. **(K)** Patients with stages I-II. **(L)** Patients with stages III-IV.

We also observed that the signature was applicable to predict the prognosis of colon cancer patients in different clinical subgroups, including different ages, different gender, different T stages, different N stages, different M stages, and different pathological TNM stages (Figures 8A–L).

### Gene mutation landscape in high- and low-risk score group

We also investigated gene mutations in different risk score groups. The gene mutation frequency of the low-risk score group was higher than the high-risk score group. The top 20 genes with mutation frequencies in the high-risk score group were visualized in Supplementary Figure S6A, while the low-risk score group was visualized in Supplementary Figure S6B.

### Immunotherapy response in high- and low-risk score groups

**FIGURE 9:**
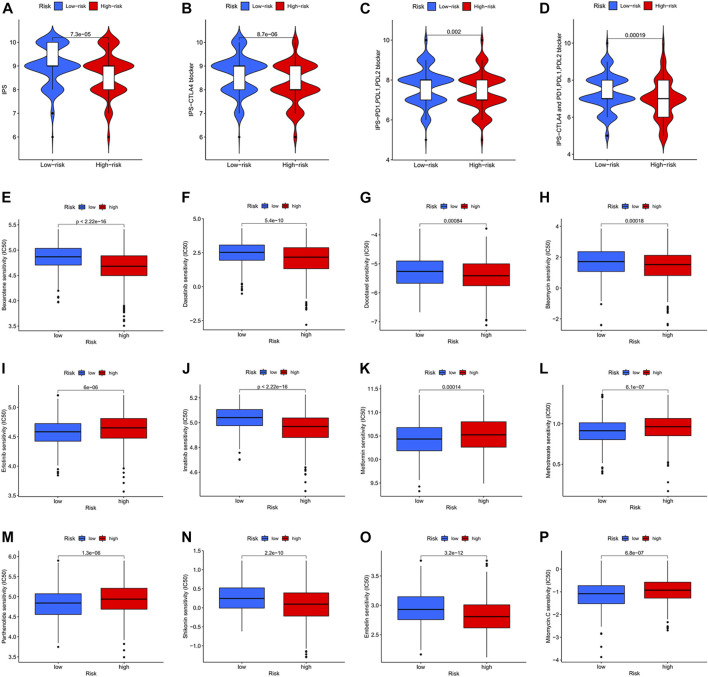
Immunotherapy response and chemotherapy response in colon cancer. **(A)** Immunotherapy response of different risk score groups for CTLA4-negative and PD1, PDL1, PDL2 negative. *p* = 7.3e-05. **(B)** Immunotherapy response of different risk score groups for CTLA4 positive and PD1, PDL1, PDL2 negative. *p* = 8.7e-06. **(C)** Immunotherapy response of different risk score groups for CTLA4 negative and PD1, PDL1, PDL2 positive. *p* = 0.002. **(D)** Immunotherapy response of different risk score groups to CTLA4 positivity and PD1, PDL1, PDL2 negativity. *p* = 0.00019. **(E–P)** Chemotherapy response of different risk score groups to 12 chemotherapy drugs.

In clinical work, patients with colon cancer have individual differences and develop different responses to different therapeutic drugs, resulting in different therapy outcomes. Immunotherapy and chemotherapy are currently the main tools in the systemic therapy of colon cancer. We observed that patients in the low-risk score group had higher immune scores (IPS) and better responses to immunotherapy drugs (CTLA4, PD1, PDL1, PDL2) (Figures 9A–D). We also identified 12 drugs suitable for colon cancer (Figures 9E–P). In particular, the IC50 values of four drugs (Erlotinib, Metformin, Methotrexate, and Mitomycin) were lower in the low-risk score group and more suitable for patients in the low-risk score group. In contrast, the IC50 values of eight drugs (Bexarotene, Bleomycin, Dasatinib, Docetaxel, Embelin, Imatinib, Pazopanib, and Shikonin) were lower in the high-risk score group and more applicable to patients in the high-risk score group.

### Validation of the signature genes in normal and tumor tissues

**FIGURE 10:**
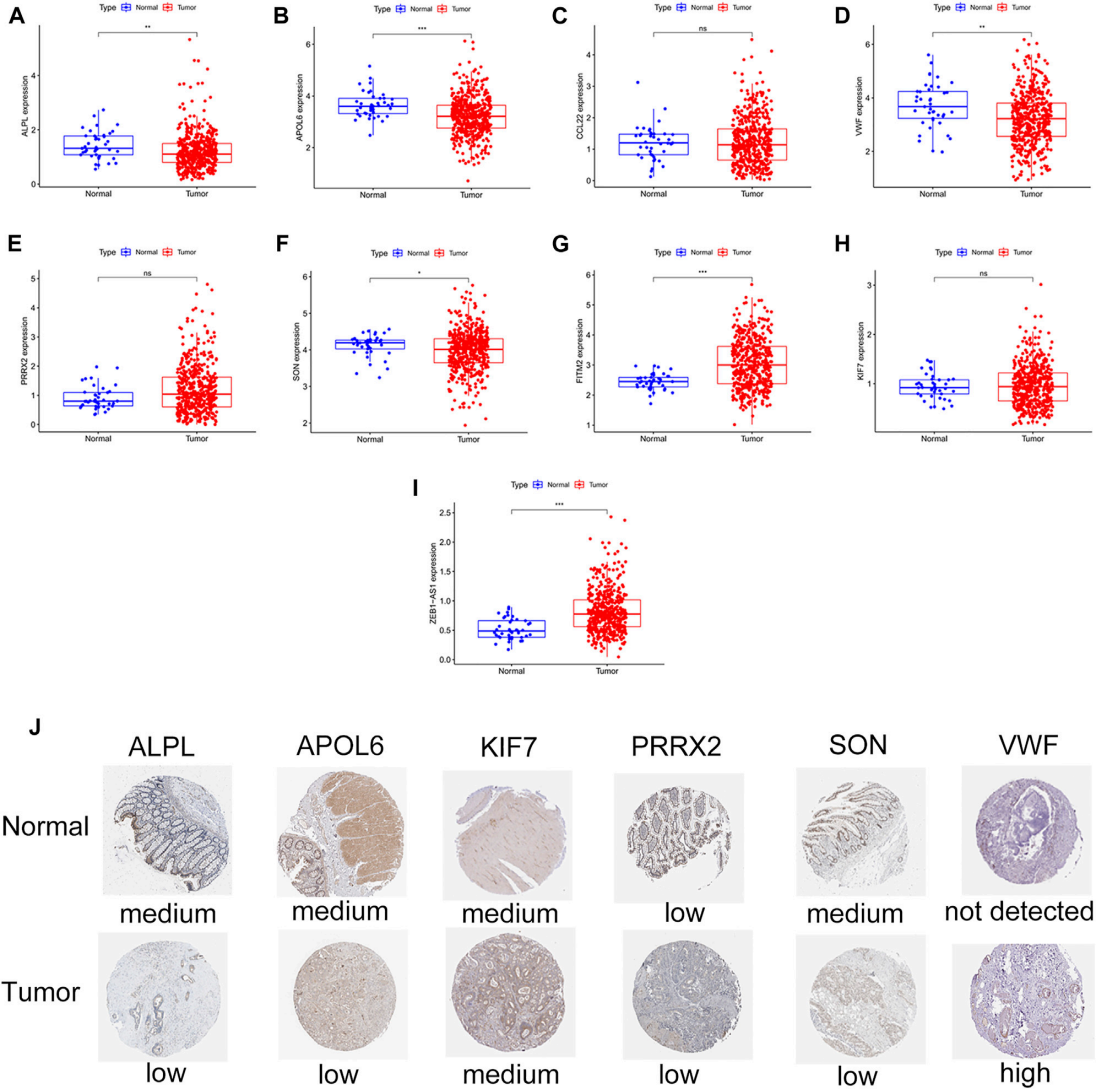
Validation of differential expression of FITM2, APOL6, VWF, PRRX2, CCL22, ALPL, SON, KIF7, and ZEB1-AS1 in normal samples and colon cancer samples. **(A–I)** Box plots. ns represents no difference; ^∗^ *p* < 0.05, ^∗^^∗^ *p* < 0.001, ^∗^^∗^^∗^ *p* < 0.001. **(J)** Immunohistochemical maps of ALPL, APOL6, KIF7, PRRX2, SON, and VWF expression proteins in the HPA database.

ALPL, APOL6, SON, VWF, FITM2, and ZEB1-AS1 were significantly differentially expressed in normal and colon cancer tissues (Figures 10A–I). In particular, ALPL, APOL6, SON, and VWF were lowly expressed in tumor tissues. In contrast, FITM2 and ZEB1-AS1 were highly expressed in tumor tissues. We also confirmed the differential expression of ALPL, APOL6, SON, and VWF in the HPA database (Figure 10J). ALPL, APOL6, SON, and VWF proteins were significantly differentially expressed between normal and tumor tissues. In contrast, the expression levels of KIF7 and PRRX2 showed no difference between normal and tumor tissues.

### Copy number variants and gene mutation in signature genes

**FIGURE 11:**
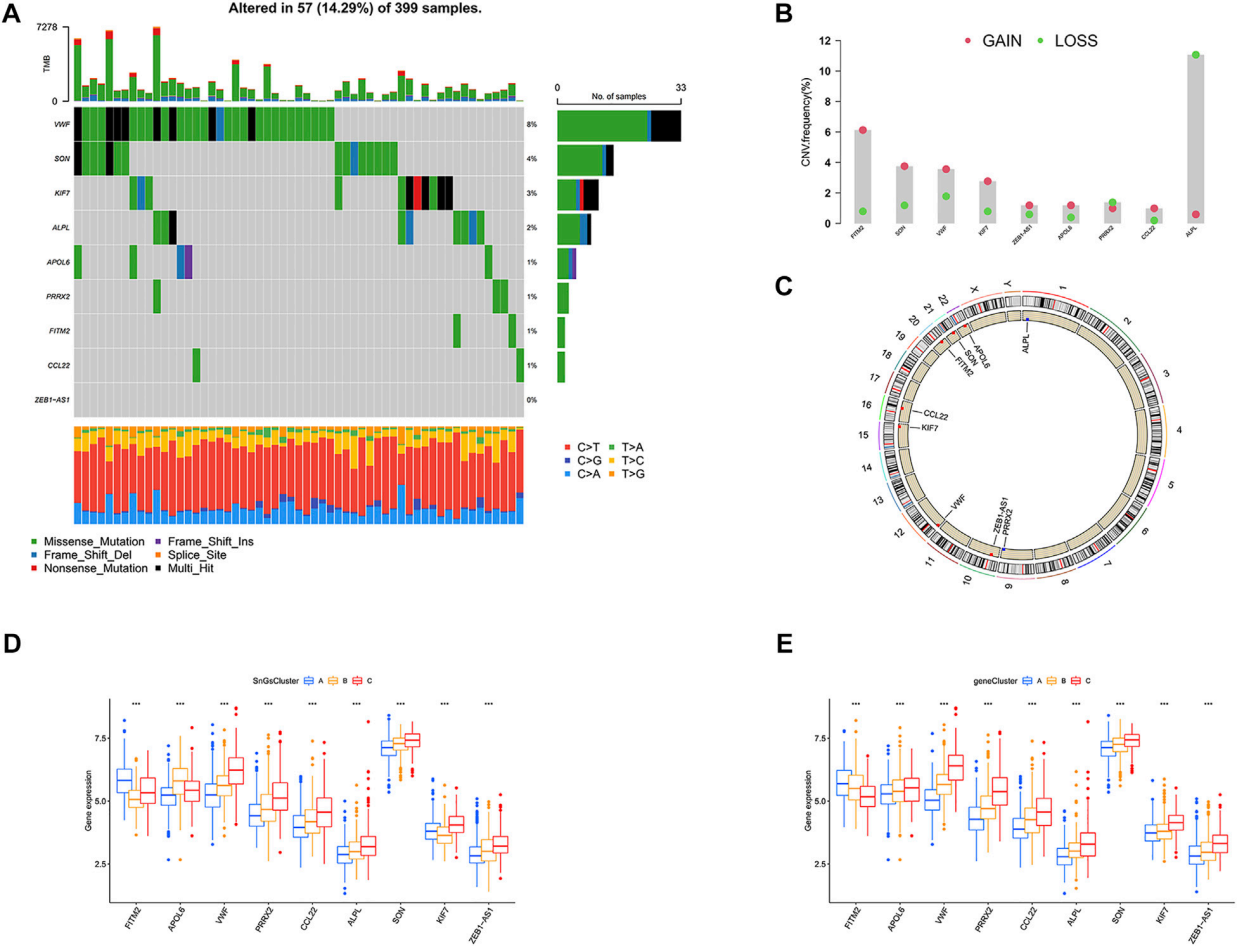
The landscape for mutation and expression of the 10 signature genes (FITM2, APOL6, VWF, PRRX2, CCL22, ALPL, SON, KIF7, and ZEB1-AS1). **(A)** The gene mutation frequency waterfall plot of 10 signature genes. **(B)** CNV alteration frequency of the 10 signature genes; Red, CNV gain; Green, CNV loss. **(C)** CNV changes in 10 signature genes on 23 chromosomes. Red, CNV increase; Blue, copy number loss. **(D)** Differential expression of 10 signature genes among three clusters. ^∗^^∗^^∗^ *p* < 0.001. **(E)** Differential expression of 10 signature genes among three gene clusters. ^∗^^∗^^∗^ *p* < 0.001.

We further investigated the gene mutation and copy number variants (CNV) for 10 signature genes in colon cancer. The gene with the highest mutation frequency was VWF at 8%, while ZEB1-AS1 had the lowest mutation frequency at 0% (Figure 11A). Interestingly, we observed that all genes showed amplification except PRRX2 and ALPL, which showed depletion (Figure 11B). And the chromosomal location of the CNV changes was visualized in Figure 11C. We also analyzed the expression of signature genes in different clusters. We observed that most of the signature genes were highly expressed in cluster C and gene Cluster C (Figures 11D,E).

## Discussion

Cellular senescence has been revealed to inhibit the progression of colon cancer cells (Acosta et al., 2013; Cho et al., 2013). However, paradoxically, cellular senescence has also been found to promote the development of colon cancer (Guo et al., 2019). It might be related to the fact that it is highly heterogeneous (Sikora et al., 2021; Wang and Demaria, 2021). In view of the importance of cellular senescence in colon cancer, we would like to construct a cellular senescence-related risk score signature to predict prognosis and immunotherapy response.

In this article, firstly, all colon cancer samples were classified into three clusters based on cellular senescence-related gene expression. Clusters A and B had a better prognosis. In contrast, cluster C showed a worse prognosis since it contained high levels of immunosuppressive cell infiltration (MDSCs, Tregs, and macrophages) (Togashi and Nishikawa, 2017; Tian et al., 2019; Katopodi et al., 2021). The reasons for the differential prognosis of the three clusters were also revealed in the results of the GSVA analysis. The enrichment pathway in cluster A was associated with anti-tumor (Kim, 2020; Wenes et al., 2022). “PARKINSONS DISEASE” and “HUNTINGTONS DISEASE” were significantly enriched in cluster B. Patients with neurodegenerative diseases were considered to be at low risk of developing cancer (Leong et al., 2021; Panegyres and Chen, 2021). In contrast, the enriched pathways in cluster C were involved in the development and metastasis of cancer (Zhao et al., 2018; Bao et al., 2019; Pudełko et al., 2019; Wu et al., 2021). Furthermore, the deficiencies of “BASE EXCISION REPAIR” and “HOMOLOGOUS RECOMBINATION” in cluster C were thought to be associated with a worse prognosis of cancer (Wallace et al., 2012; Toh and Ngeow, 2021). Next, 2334 DEGs among three clusters were identified. We observed that significantly enriched pathways in DEGs were associated with tumor development and metastasis (Bourboulia and Stetler-Stevenson, 2010; Huang et al., 2017; Izdebska et al., 2018; Li et al., 2021; Lin et al., 2022). It confirmed that cellular senescence played a crucial role in the prognosis of colon cancer. In order to further validate the above speculation, 2334 DEGs among clusters were performed consensus clustering analysis. All colon cancer patients were divided into three gene clusters. Gene cluster A had the best prognosis, while gene cluster C had the worst prognosis. It suggested that cellular senescence could also distinguish prognostic differences among gene clusters. Therefore, we considered that patients with different prognoses of colon cancer could be distinguished based on cellular senescence.

Next, according to 681 prognosis-related DEGs among clusters, a cellular senescence-related risk score signature (FITM2, APOL6, VWF, PRRX2, CCL22, ALPL, SON, KIF7, and ZEB1-AS1) was constructed to predict patients’ prognosis. Low-risk score group showed longer survival and a lower percentage of deaths. The risk score could be used independently of other clinical features (age, gender, stage, T stage, N stage, and M stage) to predict patients’ prognosis with the highest accuracy. Moreover, compared to other signatures, the cellular senescence-related risk score signature had the highest predictive accuracy with a C-index value of 0.682. Excitingly, we observed that the cellular senescence-related risk score signature predicted little heterogeneity in prognosis between the training set and testing set by performing the prognostic meta-analysis with I^2^ < 50%. It further confirmed the accuracy of the signature in predicting the prognosis of colon cancer patients. A nomogram predicting 1-year, 3-years, and 5-years survival probability in patients with colon cancer was constructed for the clinical work. It has the highest accuracy compared to other clinical characteristics, with an AUC value of 0.823. According to the comparison of risk scores in different subgroups, we observed the following phenomena: more advanced TNM stage was associated with higher risk scores; cluster C had a significantly higher risk score than cluster A and cluster B; gene cluster C had a significantly higher risk score than gene cluster A; the clinical outcome death group had a significantly higher risk score than the survival group. It further confirmed that the high-risk score group was associated with a worse prognosis, while the low-risk score group was associated with a better prognosis. There was very high accuracy in distinguishing between high and low-risk score groups based on the risk score signature. We also demonstrated the suitability of the signature for predicting prognosis in different clinical subgroups, including different age groups (≤65 and >65), different gender (female and male), different T stages (T-2 and T3-4), different N stages (N0 and N1-3), different M stages (M0 and M1), and different TNM stages (stage I-II and stage III-IV). Since it is impossible to identify which colon cancer patients benefit from immunotherapy in clinical work, which often leads to misuse of immunotherapy drugs. Therefore, we performed further analysis to explore whether the signature could distinguish colon cancer patients who have better immunotherapy responses for targeted treatment. The low-risk score group had a better immunotherapy response. While the low-risk score group had a worse immunotherapy response. Therefore, better benefits may be achieved when immunotherapeutic drugs (PD1/PDL-1/PD-L2/CTLA-4 blockers) are used for colon cancer patients in the low-risk score group.

Finally, FITM2, APOL6, VWF, PRRX2, CCL22, ALPL, SON, KIF7, and ZEB1-AS were further investigated. In our study, FITM2 was highly expressed in colon cancer tissues. It was consistent with the findings of Yang et al. (Yang et al., 2019). We demonstrated that TITM2 was a low-risk gene, which was associated with a better prognosis in patients with colon cancer. APOL6 showed a low expression level in colon cancer tissues and was a low-risk gene. It was due to the ability of APOL6 to induce apoptosis in colon cancer cells (Aryee et al., 2013). In our article, VWF was low expressed in colon cancer tissue and was a high-risk gene. It was because VWF could promote a highly aggressive nature of colon cancer (Zanetta et al., 2000). Our study showed that PRRX2 was a high-risk gene. However, Chai et al. considered that PRRX2 inhibited distant metastasis of colon cancer cells and was a protective gene (Chai et al., 2019). This difference required further verification in subsequent experiments. Chen et al. revealed that high expression of CCL22 was associated with a better prognosis in patients with colon cancer (Chen et al., 2021). It was consistent with our findings. Luo et al. found that ALPL inhibited the aggressiveness of ovarian cancer (Luo et al., 2019). And Child et al. identified ALPL as a cancer suppressor gene for prostate cancer (Tong et al., 2019). However, the opposite was true for the role of ALPL in colon cancer. In our study, ALPL was a high-risk gene that was lowly expressed in colon cancer tissues. The significance of SON in colon cancer has not been studied by anyone. We first identified SON as a high-risk gene with low expression in colon cancer tissues. Hu et al. revealed that downregulation of KIF7 promoted antitumor activity in lung cancer and it was a cancer-promoting gene (Hu et al., 2020). Surprisingly, we also found KIF7 as a high-risk gene in colon cancer. In our article, ZEB1-AS1 was highly expressed in colon cancer tissues and was associated with a worse prognosis. This was associated with the ability of ZEB1-AS1 to cause the malignant progression of colon cancer (Ni et al., 2020). We also found that VWF had the highest mutation frequency, while ZEB1-AS1 was not mutated. All genes showed amplification except for PRRX2 and ALPL which showed depletion. Most of the signature genes were upregulated in cluster C and gene cluster C.

In conclusion, the cellular senescence-related risk score signature could be used to predict prognosis and immunotherapy response in colon cancer.

## Data Availability

Publicly available datasets were analyzed in this study. The names of the repository/repositories and accession number(s) can be found in the article/Supplementary Material.

## Supplementary material

Supplementary Figure S1 The design flow chart for the research.

Supplementary Figure S2 Clustering results based on the expression of 279 cellular senescence-related genes. Cluster Num = 2, 4, 5, 6, 7, 8, 9. [Not Available in CrossRef].

Supplementary Figure S3 Identification of DEGs in clusters. **(A)** Venn diagram. 2334 DEGs were acquired by taking intersections of cluster AB, cluster AC, and cluster BC differential genes. **(B)** Bubble diagram of GO enrichment. BP, biological process; MF, molecular function; CC, cellular component; vertical coordinates represent GO names, horizontal coordinates represent gene proportions; circle size represents the number of genes enriched on GO; circle color represents enrichment significance. **(C)** KEGG enrichment bubble diagram. Vertical coordinates represent GO names, horizontal coordinates represent gene proportions; circle size represents the number of genes enriched on GO; circle color represents enrichment significance.

Supplementary Figure S4 Clustering results based on the expression of 681 prognostic DEGs among clusters. Cluster Num = 2, 3, 4, 5, 6, 7, 8, 9. When cluster Num = 3, the relationship in the gene cluster was tight and the correlation between gene clusters was weak.

Supplementary Figure S5
**(A–D)** ROC curves for the risk score signature of Ren, Wang, Zhang, and Zheng for predicting 1-, three- and 5-years survival in patients with colon cancer. **(E–H)** Patients with colon cancer were classified into high and low-risk score groups based on the risk score signature of Ren, Wang, Zhang, and Zheng. Patients in the low-risk score group had a better prognosis.

Supplementary Figure S6 Waterfall plots of gene mutations in different risk score groups. **(A)** High-risk score group. **(B)** Low-risk score group.

Supplementary Table S1 279 cellular senescence-related genes were acquired from the CellAge database.

Supplementary Table S2 2334 DEGs among Cluster A, Cluster B, and Cluster C.

Supplementary Table S3 681 prognostic DEGs among Cluster A, Cluster B, and Cluster C.

Supplementary Table S4 The statistics of clinical information for all colon cancer samples, the training set, and the testing set.
