# Supplementary figures and images for: Identification of a novel cellular senescence-related signature for the prediction of prognosis and immunotherapy response in colon cancer
Source: Front Genet. 2022 Aug 4;13:961554. doi: 10.3389/fgene.2022.961554 (PMC9386482; doi:10.3389/fgene.2022.961554)

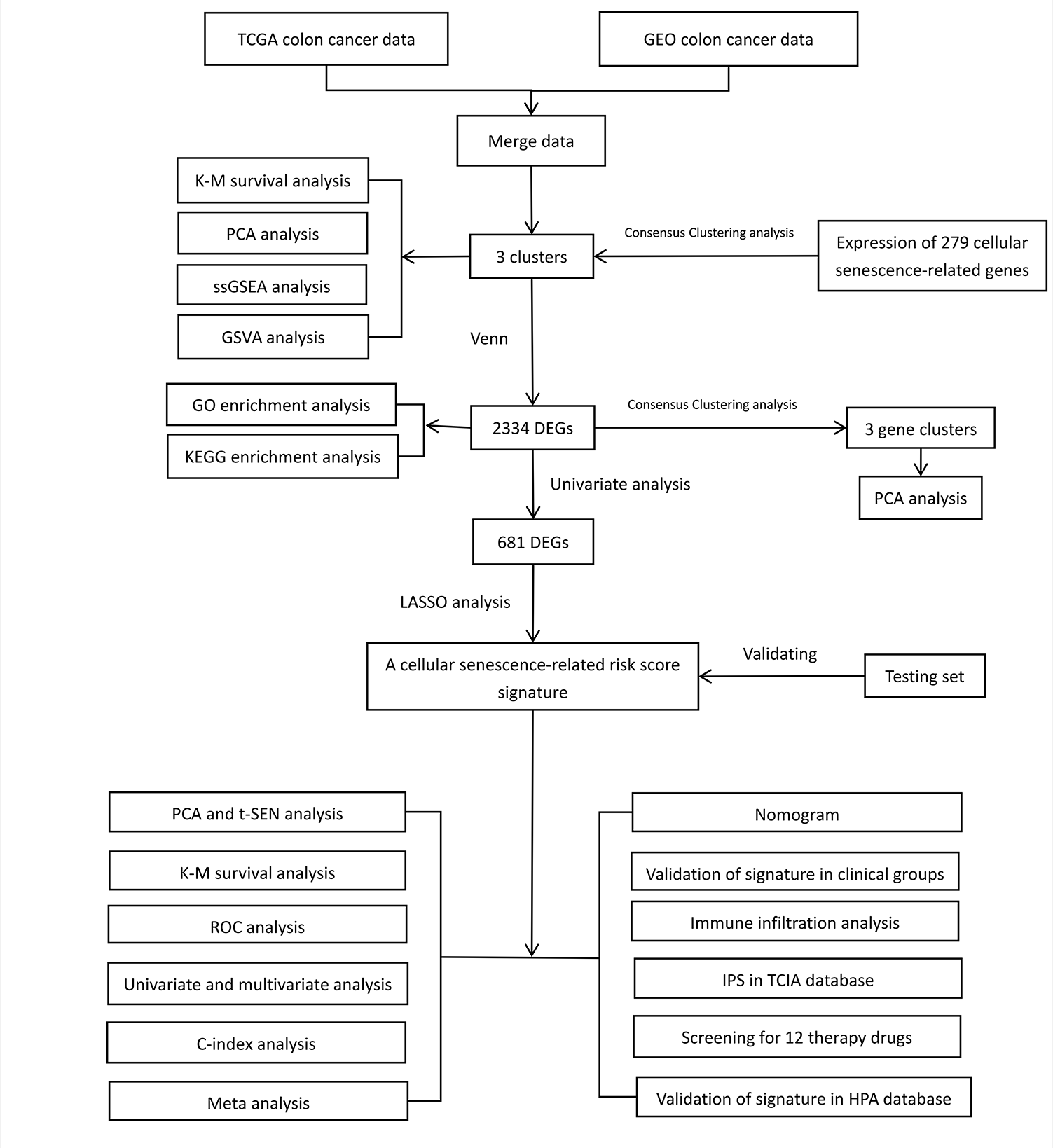

Supplement: Supplementary file 1 [file Datasheet1.zip › Supplementary materials/Supplementary Figure S1.tif]

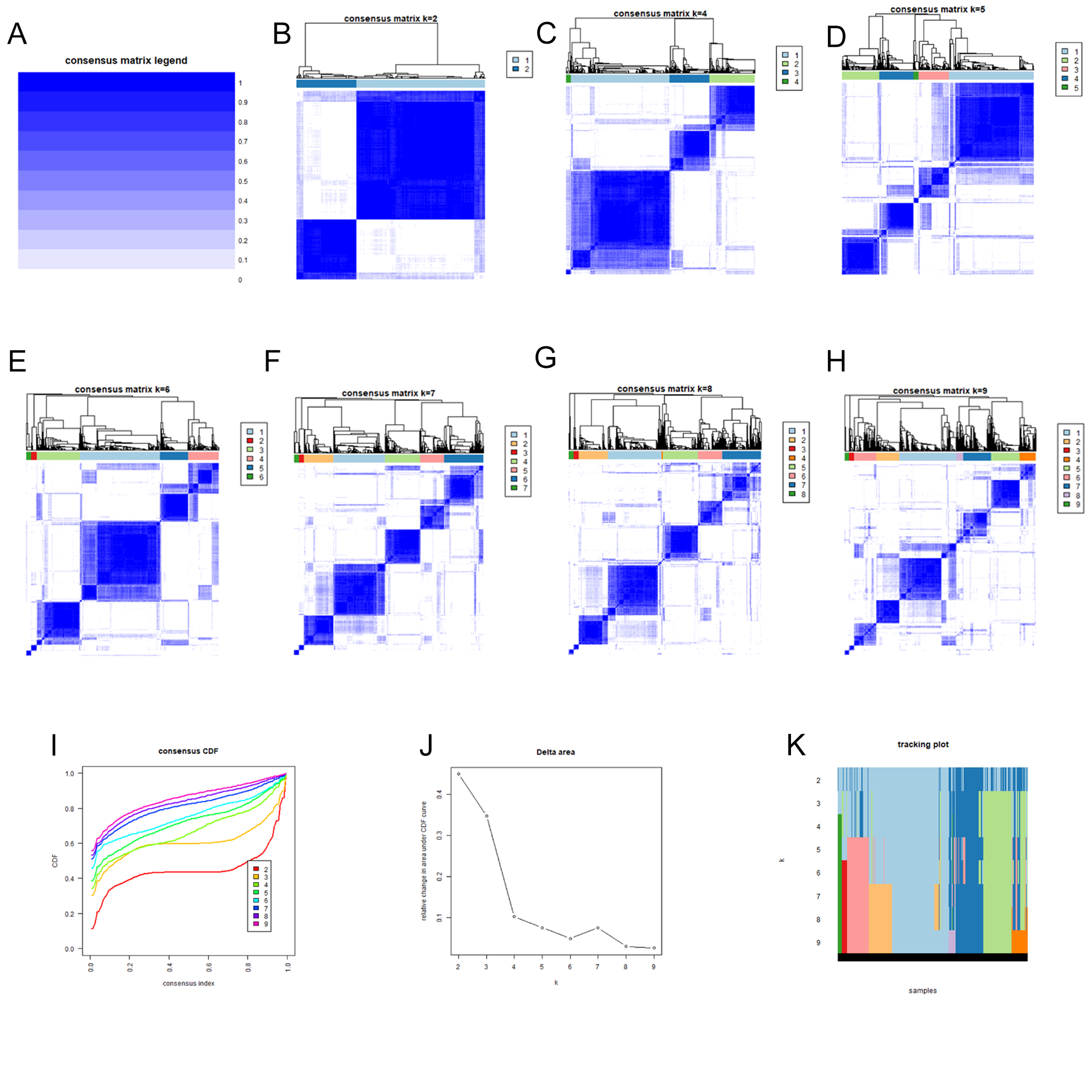

Supplement: Supplementary file 1 [file Datasheet1.zip › Supplementary materials/Supplementary Figure S2.tif]

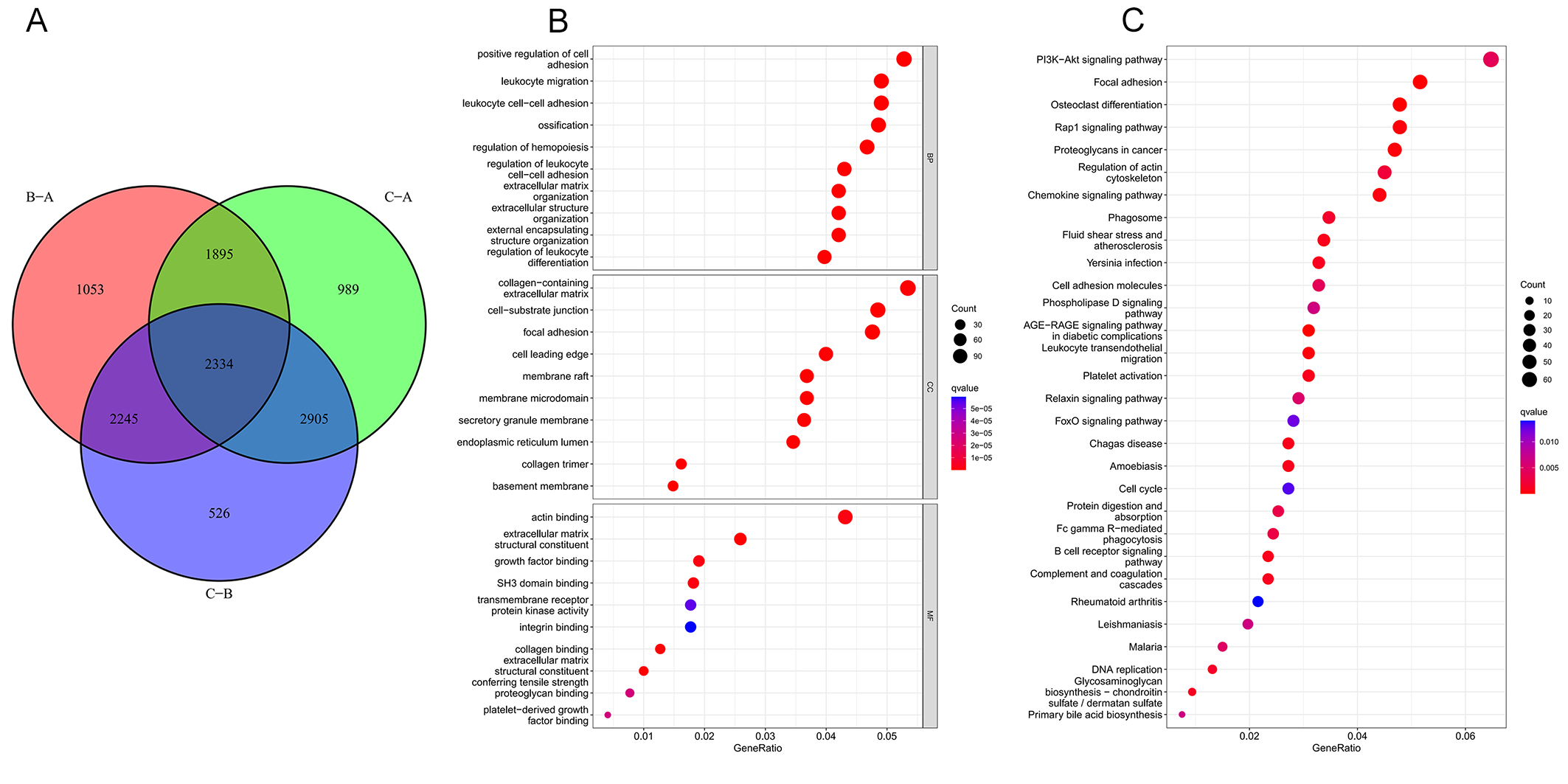

Supplement: Supplementary file 1 [file Datasheet1.zip › Supplementary materials/Supplementary Figure S3.tif]

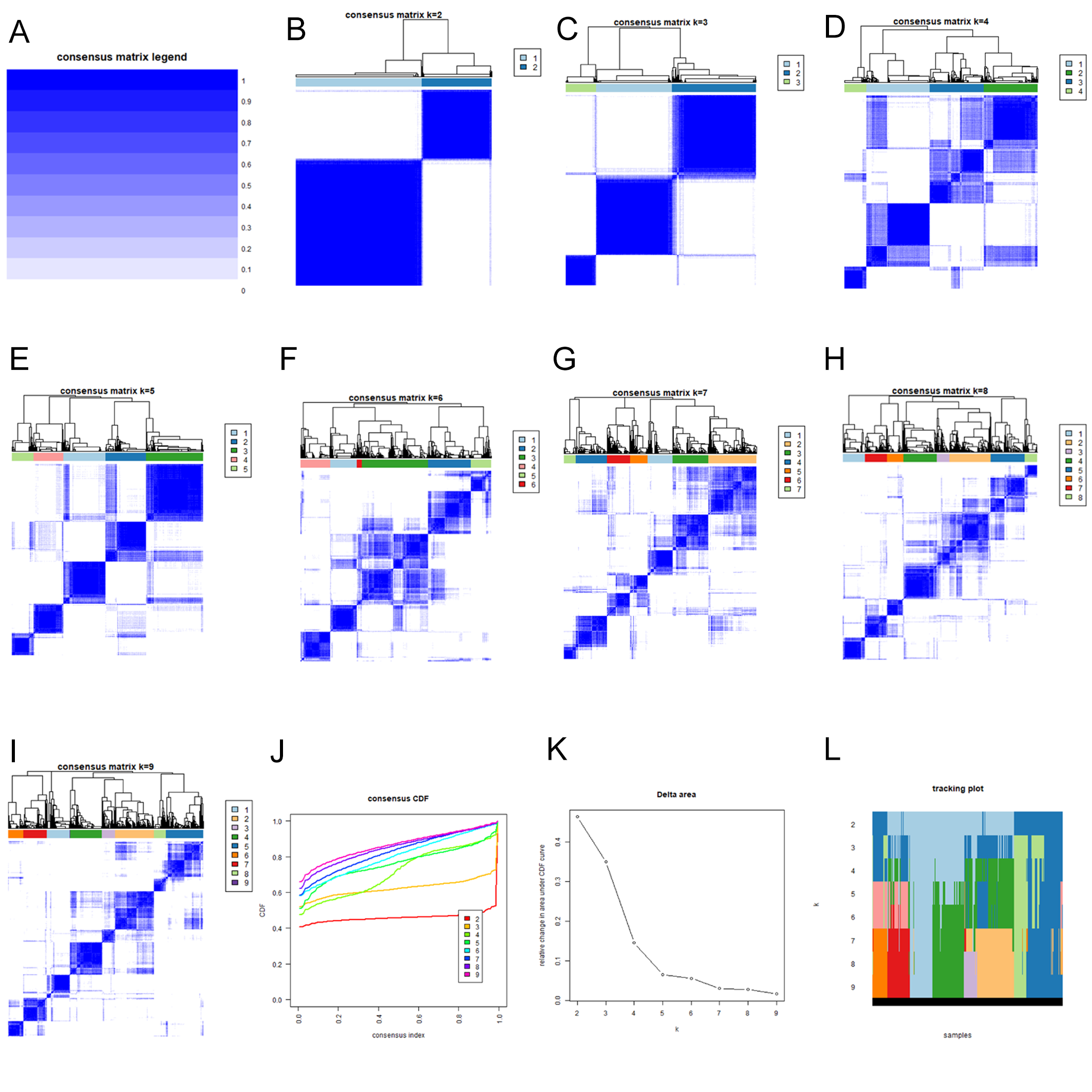

Supplement: Supplementary file 1 [file Datasheet1.zip › Supplementary materials/Supplementary Figure S4.tif]

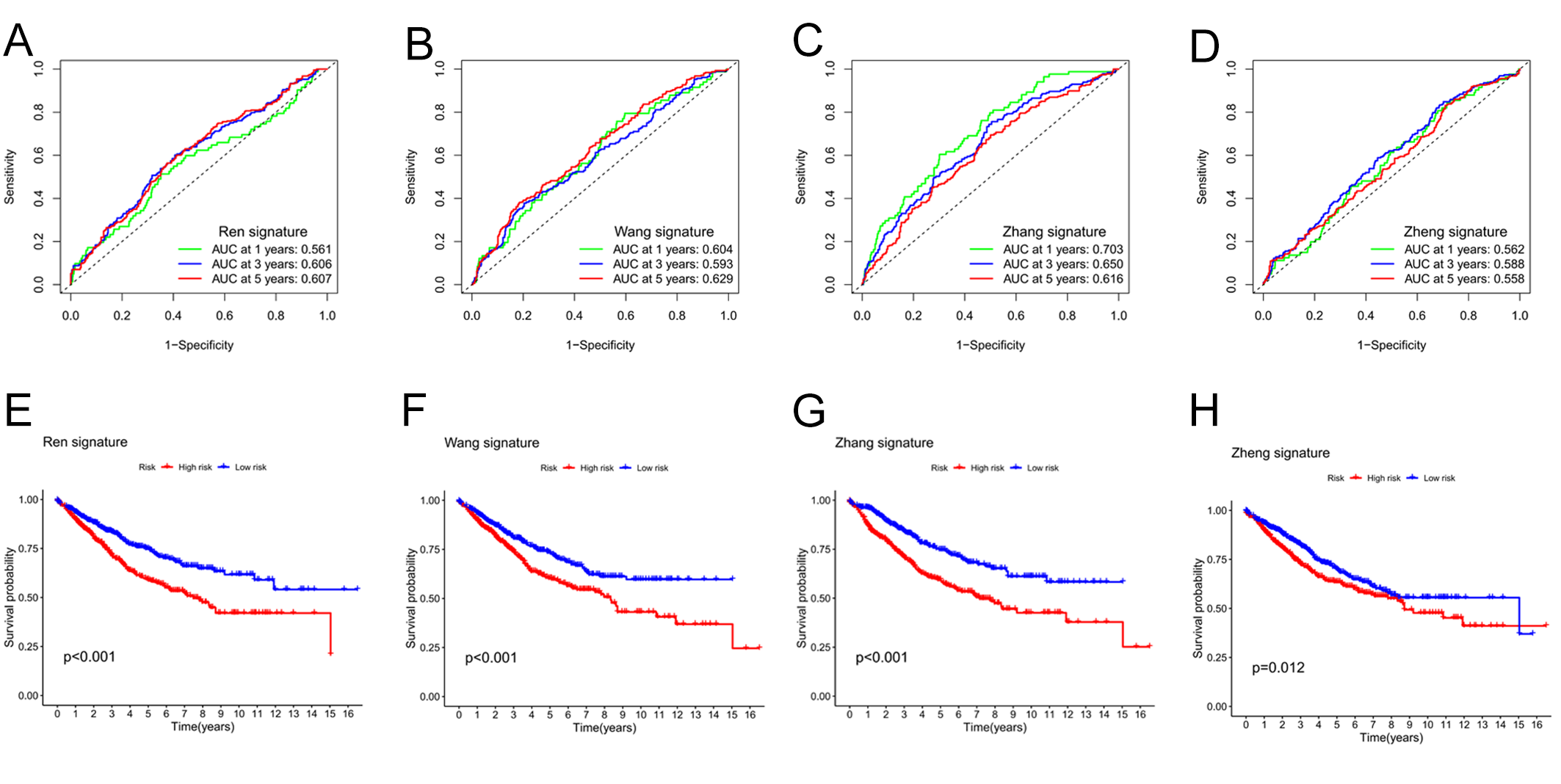

Supplement: Supplementary file 1 [file Datasheet1.zip › Supplementary materials/Supplementary Figure S5.tif]

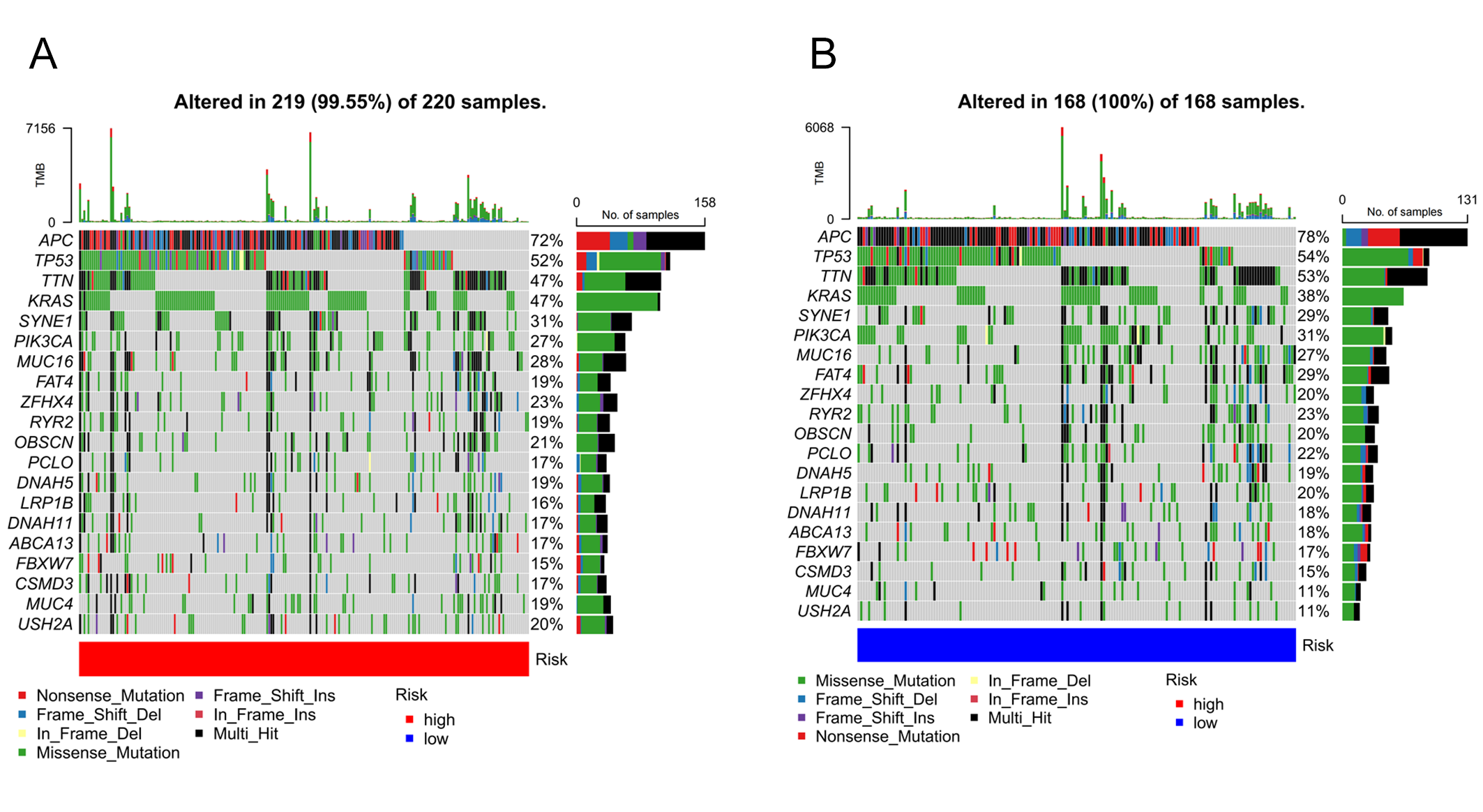

Supplement: Supplementary file 1 [file Datasheet1.zip › Supplementary materials/Supplementary Figure S6.tif]
